# Supplementary material for: Impact of pulmonary African trypanosomes on the immunology and function of the lung
Source: Nat Commun. 2022 Nov 18;13:7083. doi: 10.1038/s41467-022-34757-w (PMC9674601; doi:10.1038/s41467-022-34757-w)
Supplement: Supplementary file 1 — Supplementary Information [file 41467_2022_34757_MOESM1_ESM.pdf]

Supplementary information

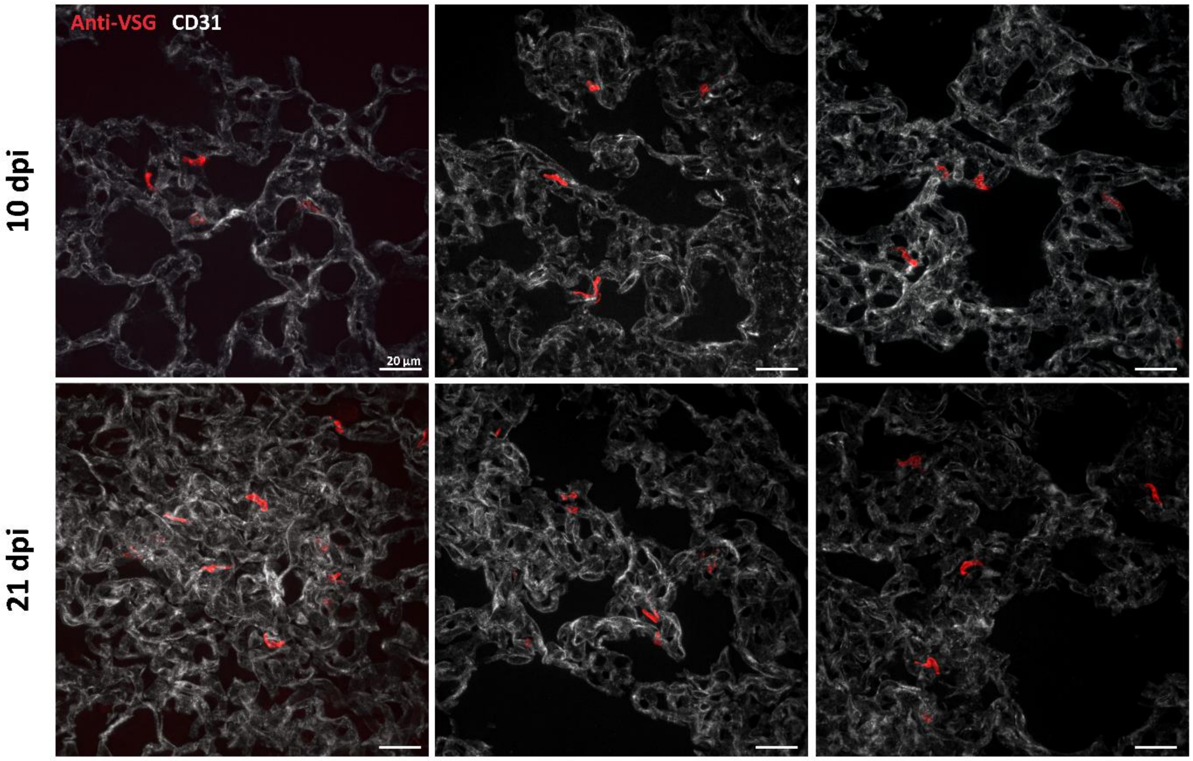

Fig. S1 Fluorescence microscopic evaluation of *T. b. brucei* infection in the lungs. Groups of three C57BL/6JRj mice were infected via the bite of a *T. b. brucei* AnTar1 infected *G. morsitans* tsetse fly. After perfusion of the vascular system and fixation via intratracheal instillation at 10 and 21 dpi, lungs were collected *en bloc* and the left lobes were embedded in PELCO cryo-embedding compound prior to cryosectioning. Sections were stained to detect the VSG-coat of the parasite (Cy3 - red) and CD31 of the endothelial cells (Cy 5 - white). Scale bar size is 20 µm.

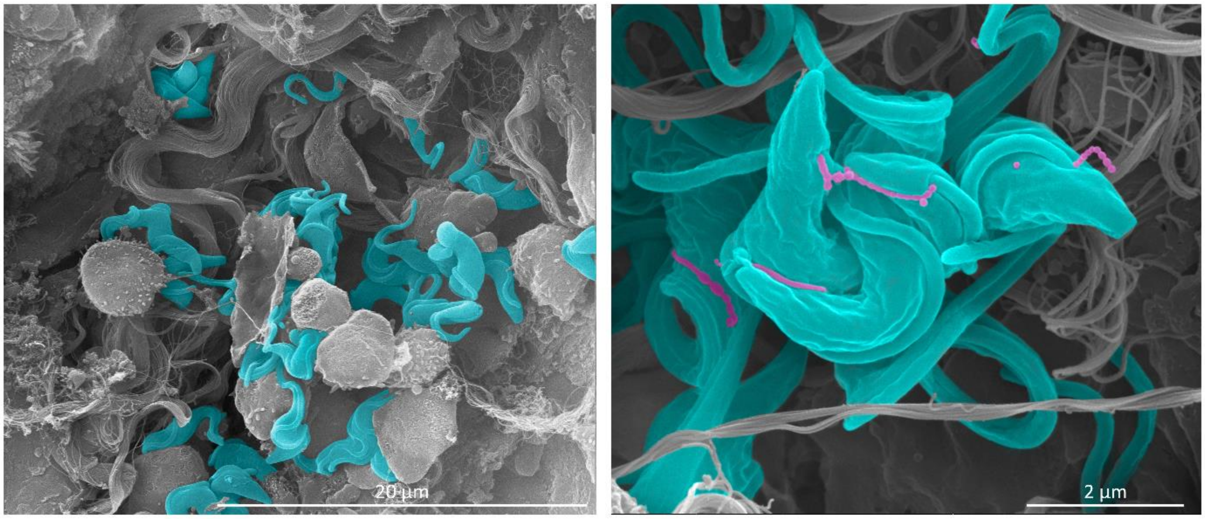

Fig. S2 False-coloured scanning electron microscopic images of lungs tissue of *T. b. brucei* (blue) infected mice. Groups of three C57BL/6JRj mice were infected via the bite of a *T. b. brucei* AnTar1 infected *G. morsitans* tsetse fly. After perfusion of the vascular system at 10 dpi, lungs were fixed in glutaraldehyde and embedded in 1% agarose prior to vibratome sectioning. Parasite interaction via the release of extracellular vesicles (pink).

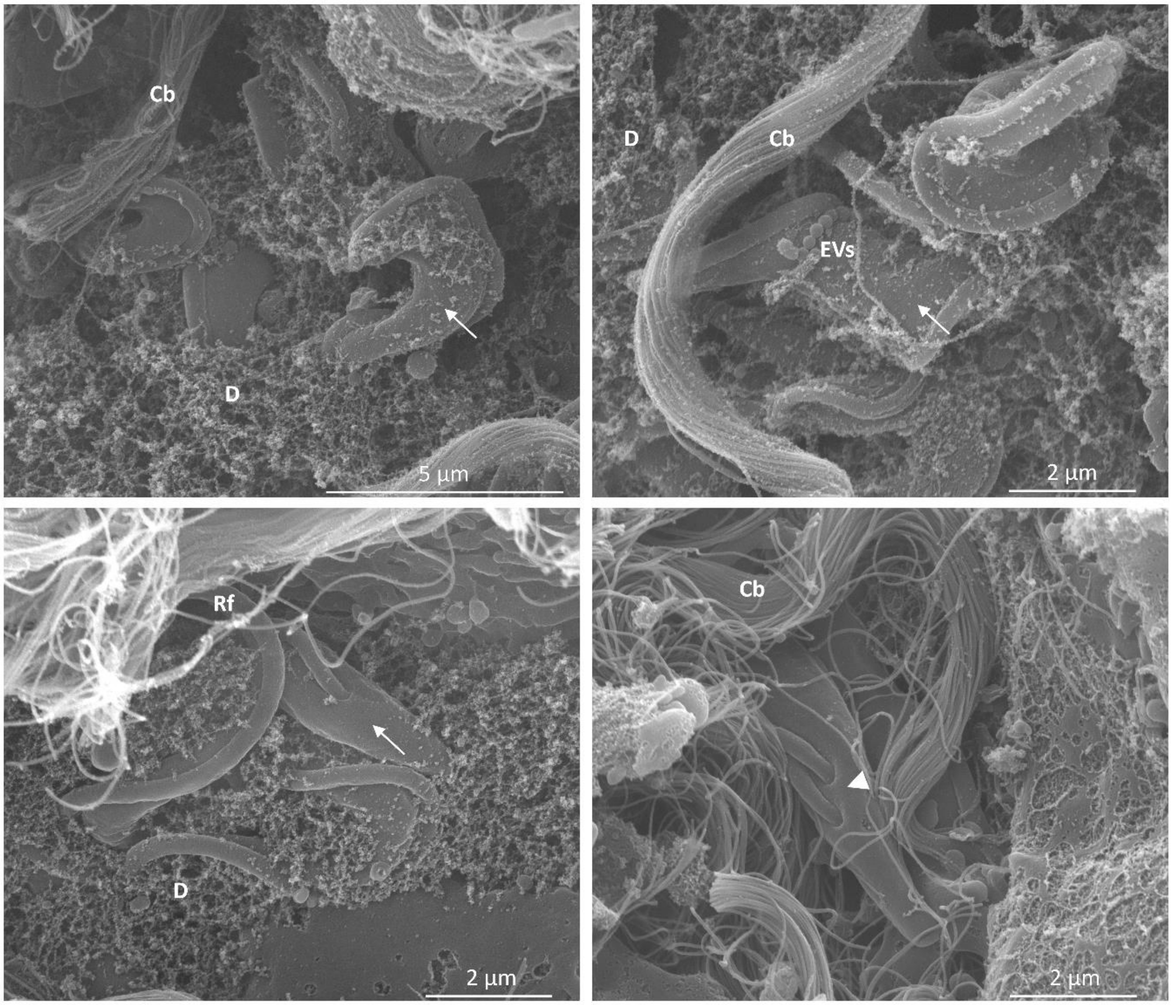

Fig. S3 Scanning electron microscopic images of the dense connective tissue in the lungs of *T. b. brucei* infected mice. Groups of three C57BL/6JRj mice were infected via the bite of a *T. b. brucei* AnTar1 infected *G. morsitans* tsetse fly. After perfusion of the vascular system at 10 dpi, lungs were fixed in glutaraldehyde and embedded in 1% agarose prior to vibratome sectioning. Cb: collagen bundle, Rf: reticular fibre, EV: extracellular vesicles, D: dense connective tissue, arrows: *T. b. brucei* parasites, arrowheads: multiplying parasites with two flagella.

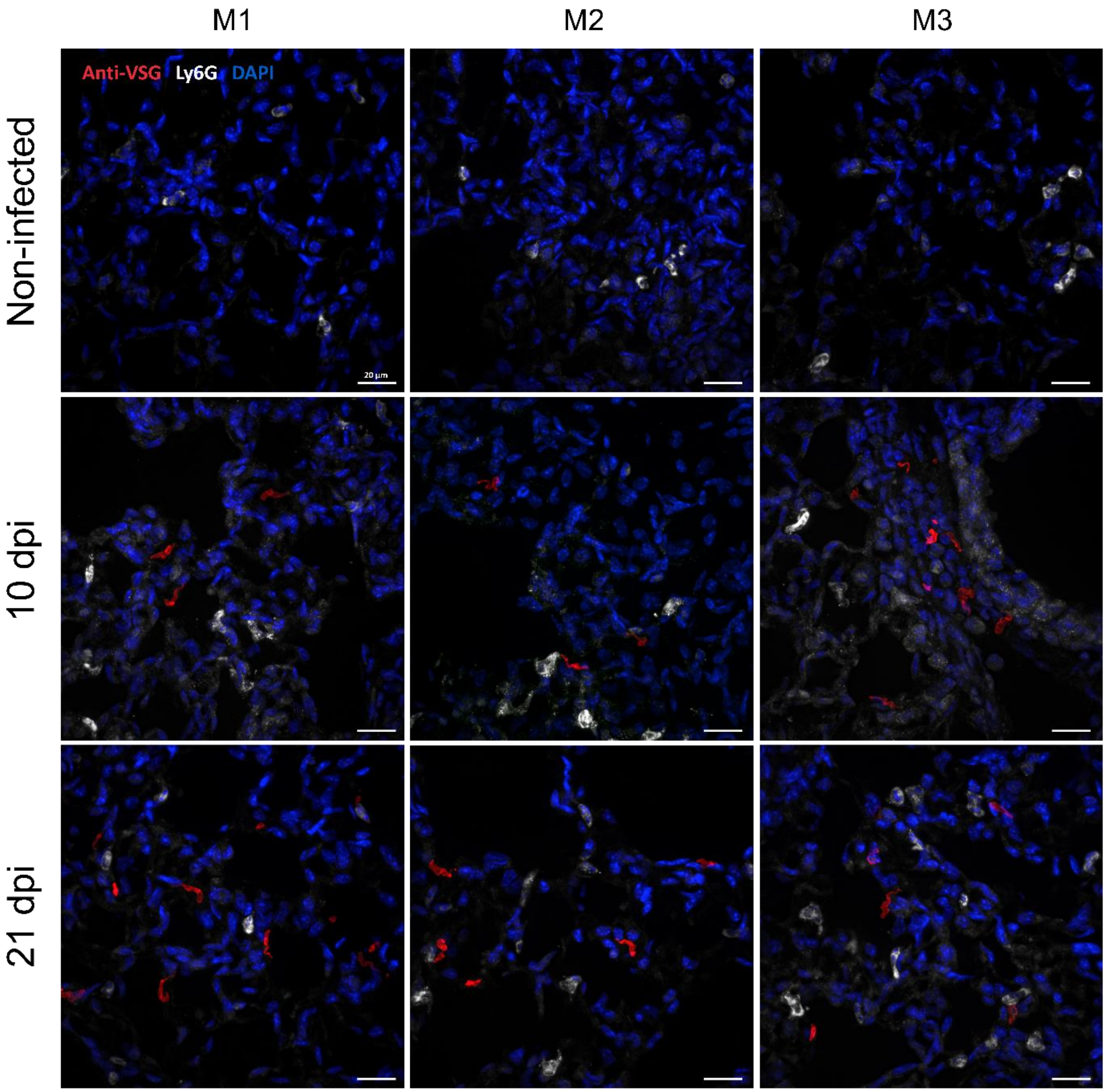

Fig. S4 Fluorescence microscopic evaluation of neutrophil presence in lungs. Groups of three C57BL/6JRj mice were infected via the bite of a *T. b. brucei* AnTar1 infected *G. morsitans* tsetse fly. After perfusion of the vascular system and fixation of the lungs via intratracheal instillation at 10 and 21 dpi, lungs were collected *en bloc* and the left lobes were embedded in PELCO cryo-embedding compound prior to cryosectioning. Sections were stained with a polyclonal antibody staining the variant surface glycoprotein (VSG) coat of the parasite (Cy3 - red), DAPI (blue) and Ly-6G as a specific marker for neutrophils (Cy5 - white). Scale bar size is 20 µm.

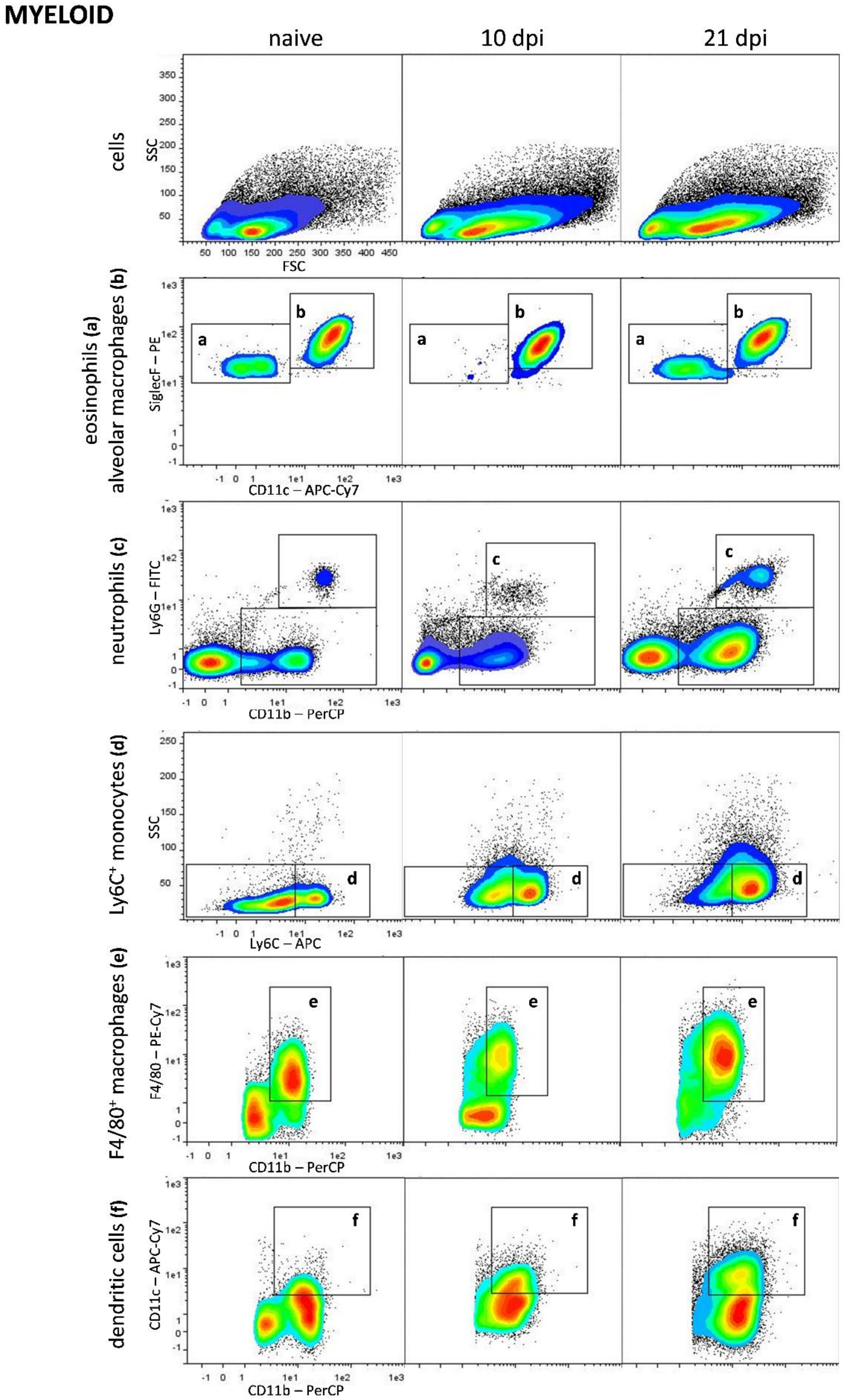

Fig. S5 Flow cytometry plots of myeloid cells in infected lung tissue. A cell gate was set in an SSC-A versus FSC-A plot, followed by gating on singlets in a FSC-H versus FSC-A plot. Live ($DAPI^-$) $CD45^+$ cells were divided into two

fractions: $SiglecF^+$ cells were further characterized as (**a**) eosinophils ($CD11c^{lo/-}$) and (**b**) alveolar macrophages ($CD11c^+$) and $SiglecF^-$ cells were further identified as (**c**) neutrophils ($CD11b^+$, $Ly\text{-}6G^+$), (**d**) monocytes ($CD11b^+$, $Ly6G^-$, SSC^{lo}, $Ly6C^{hi}$), (**e**) macrophages ($CD11b^+$, $Ly6G^-$, SSC^{lo}, $Ly6C^-$, $F4/80^+$) and (**f**) dendritic cells ($CD11b^+$, $Ly6G^-$, $CD11c^+$).

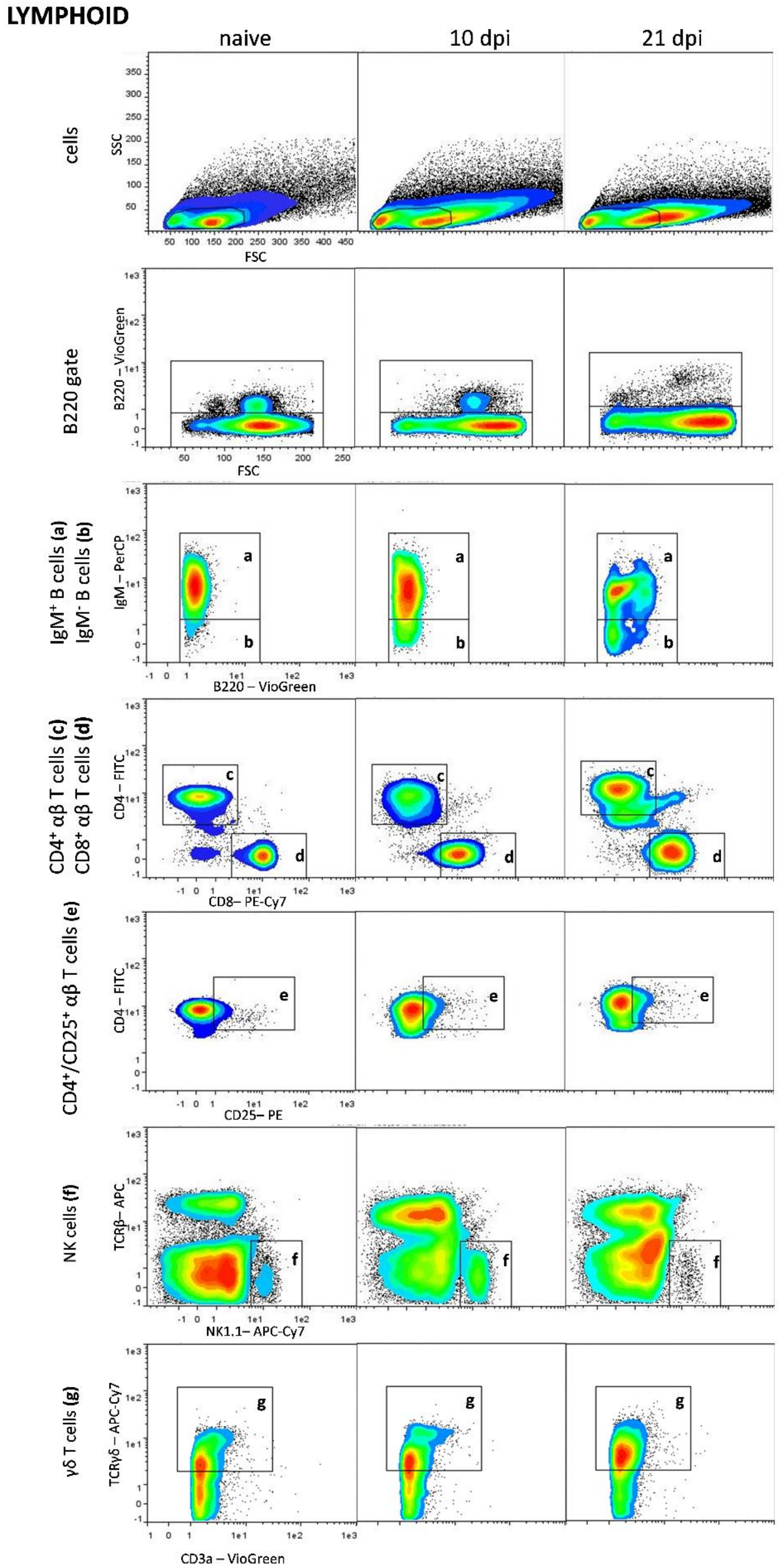

Fig. S6 Flow cytometry plots of lymphoid cells in infected lung tissue. A cell gate was set in a SSC-A versus FSC-A plot, followed by gating on singlets in a FSC-H versus FSC-A plot. Live (DAPI$^-$) cells were divided into two

fractions: B220^{+} cells were further characterized as (**a**) IgM^{+} B cells (IgM^{+}) and (**b**) IgM^{-} B cells (IgM^{-}) and B220^{-} cells were further identified as (**c**) CD4^{+} αβ T cells (TCRβ^{+}, CD8^{-}, CD4^{+}), (**d**) CD8^{+} αβ T cells (TCRβ^{+}, CD8^{+}), (**e**) CD4^{+}/CD25^{+} αβ T cells (TCRβ^{+}, CD8^{-}, CD4^{+}, CD25^{+}), (**f**) NK cells (TCRβ^{-}, NK1.1^{+}) and (**g**) CD3^{+} γδ T cells (CD3a^{+}, CD4^{-}, CD8^{-}, TCRγδ^{+}).

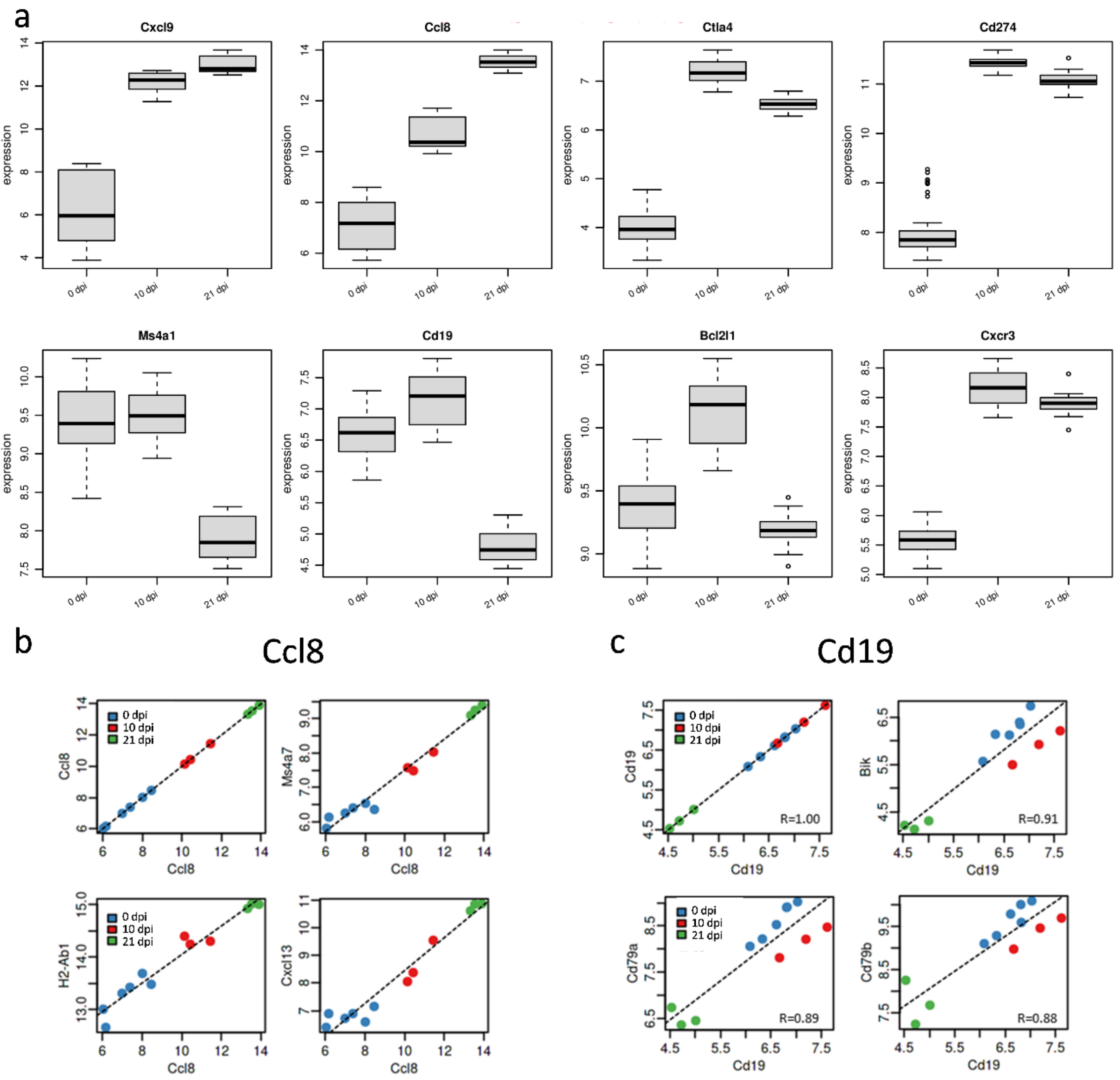

Fig. S7. Biomarker selection for the identification of *T. b. brucei* infection in the lungs. **a** Boxplots (25th percentile – median – 75th percentile) with whiskers (smallest to largest value) showing the expression of biomarker genes across the groups (NI: n=6, 10 dpi: n=3 and 21 dpi: n=3). **b,c** Scatter plots of gene expression of the top 4 correlated genes for (**b**) *Ccl8* and (**c**) *Cd19*.

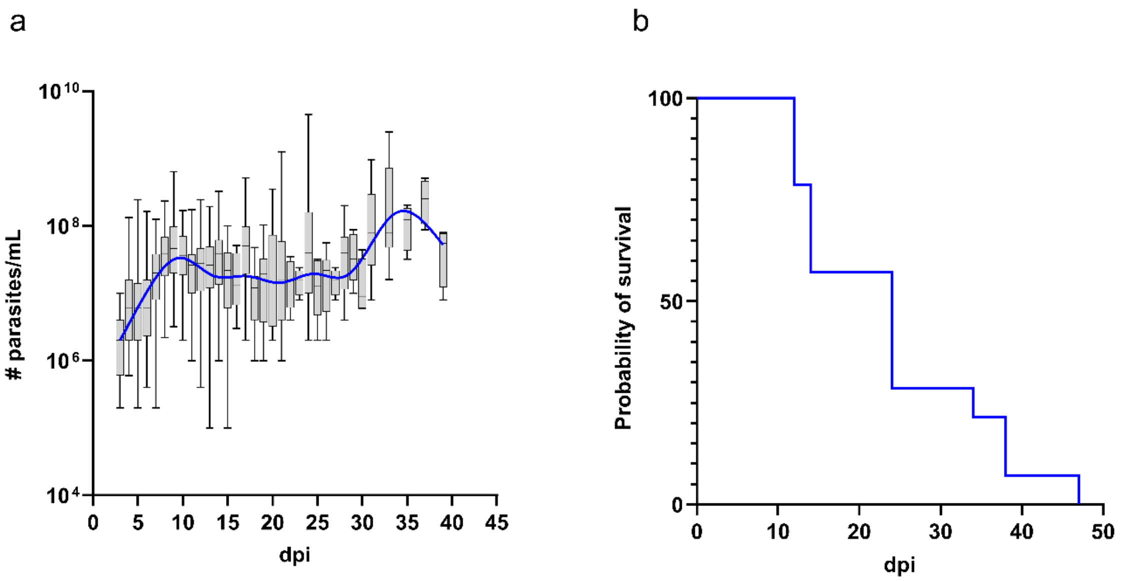

Fig. S8 Parasitemia (**a**) and survival data (**b**) for the *G. morsitans*-transmitted *T. brucei* model in C57BL/6JRj mice. **a** Blood parasitemia counts of 106 mice. The data are represented as box plots (25th percentile – median – 75th percentile) with whiskers (smallest to largest value).

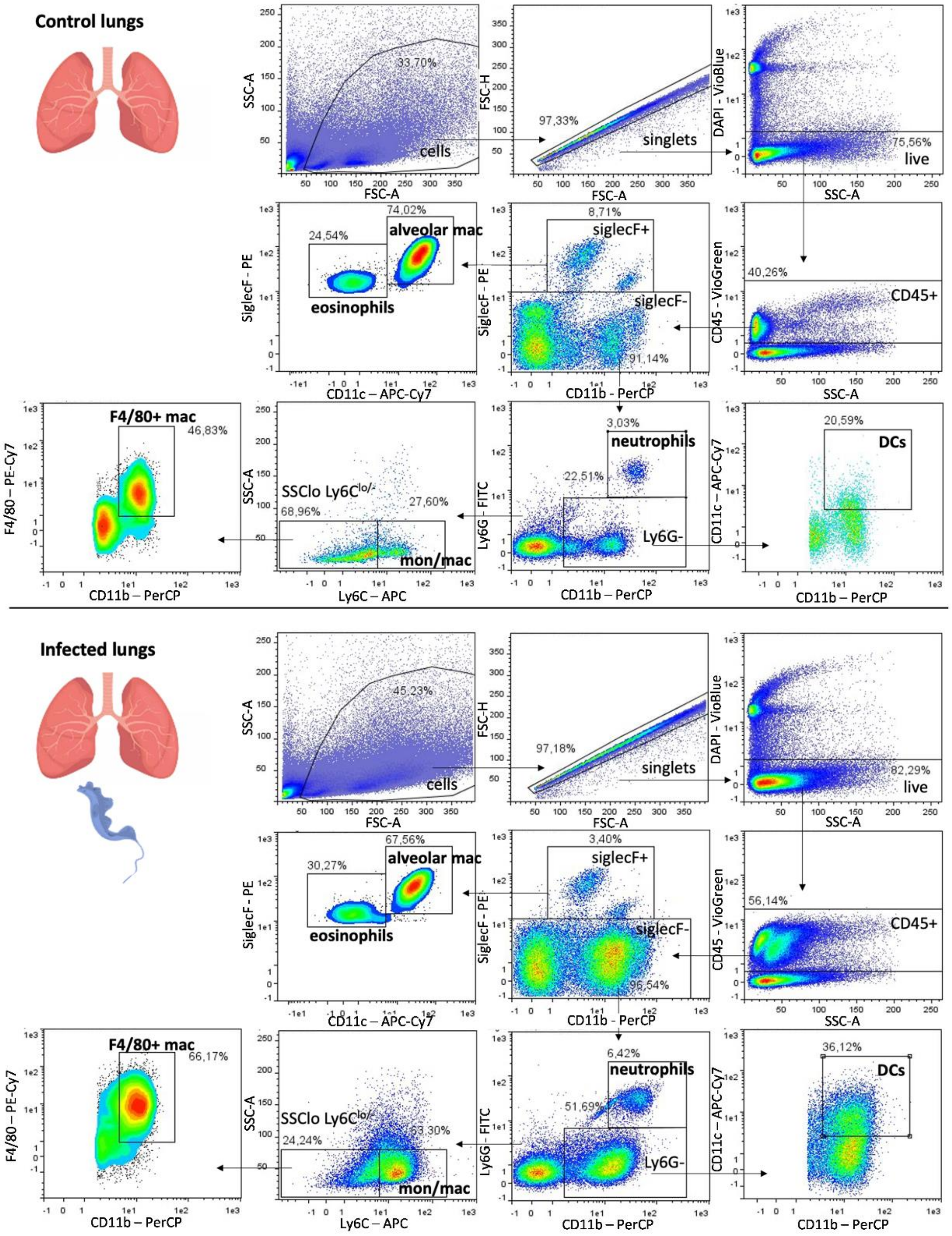

Fig. S9 Flow cytometry gating strategy for myeloid cells. A cell gate was set in an SSC-A versus FSC-A plot, followed by gating on singlets in an FSC-H versus FSC-A plot. Live (DAPI$^-$) CD45$^+$ cells were divided into two fractions: SiglecF$^+$ cells were further characterized as alveolar macrophages (CD11c$^+$) and eosinophils (CD11c$^{lo/-}$) and SiglecF$^-$ cells were further identified as neutrophils (CD11b$^+$, Ly-6G$^+$), monocytes (CD11b$^+$, Ly6G$^-$, SSC^{lo}, Ly6C^{hi}),

macrophages (CD11b^{+}, Ly6G^{-}, SSC^{lo}, Ly6C^{-}, F4/80^{+}) and dendritic cells (CD11b^{+}, Ly6G^{-}, CD11c^{+}). The gating strategy is shown in control and infected lungs (21 dpi). Parts of the figure were prepared by using clipart from Servier Medical Art. Servier Medical Art by Servier is licensed under a Creative Commons Attribution 3.0 Unported License (https://creativecommons.org/licenses/by/3.0/).

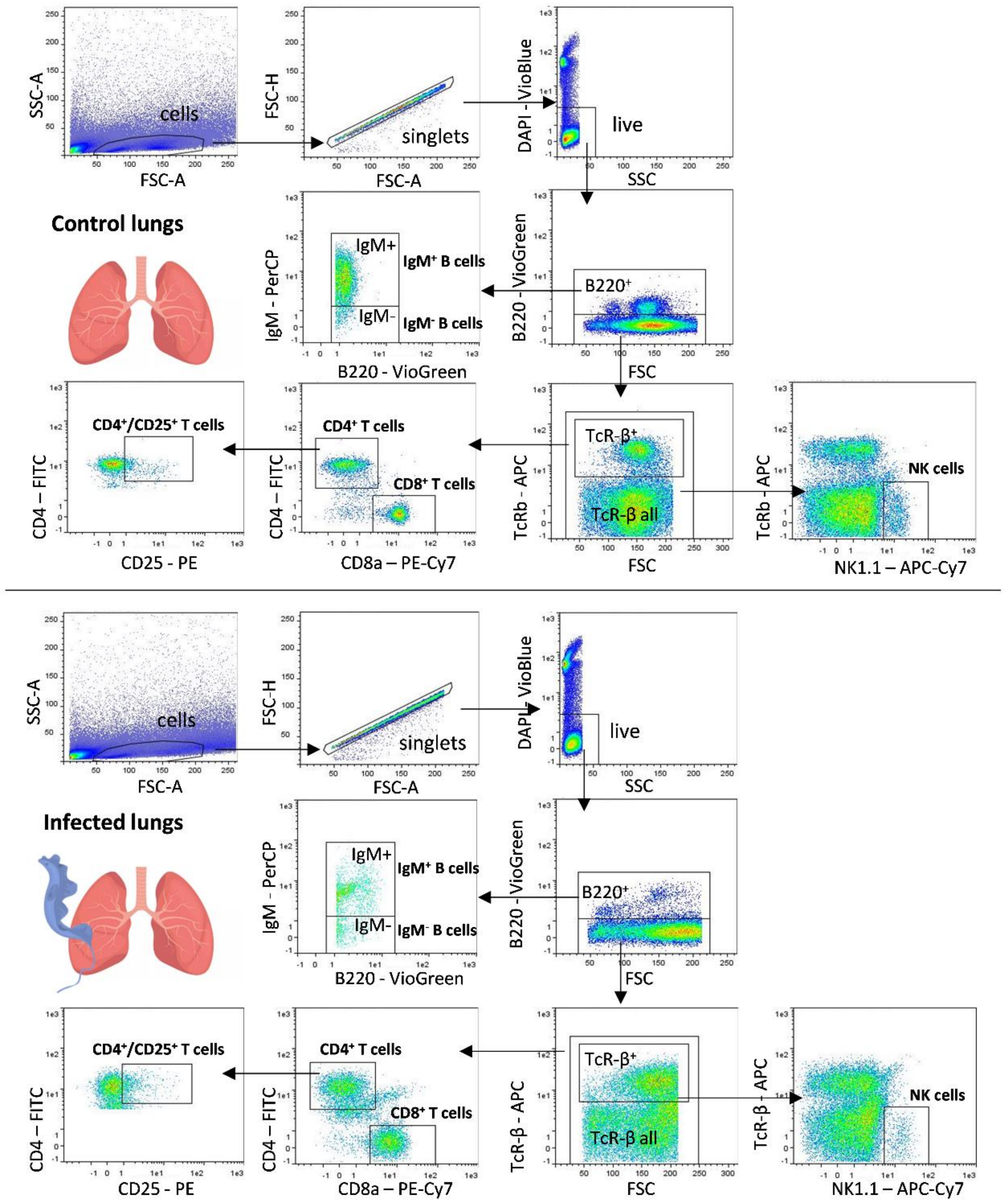

Fig. S10 Flow cytometry gating strategy for lymphoid cells. A cell gate was set in an SSC-A versus FSC-A plot, followed by gating on singlets in a FSC-H versus FSC-A plot. Live ($DAPI^-$) cells were divided into two fractions: $B220^+$ cells were further characterized as IgM^+ B cells (IgM^+) and IgM^- B cells (IgM^-) and $B220^-$ cells were further identified as natural killer cells ($TCR\beta^-$, $NK1.1^+$), $CD8^+$ αβ T cells ($TCR\beta^+$, $CD8^+$), $CD4^+$ αβ T cells ($TCR\beta^+$, $CD8^-$ $CD4^+$) and $CD4^+$/$CD25^+$ αβ T cells ($TCR\beta^+$, $CD8^-$, $CD4^+$, $CD25^+$). The gating strategy is shown in control and infected lungs (21 dpi). Parts of the figure were prepared by using clipart from Servier Medical Art. Servier Medical

Art by Servier is licensed under a Creative Commons Attribution 3.0 Unported License (https://creativecommons.org/licenses/by/3.0/).

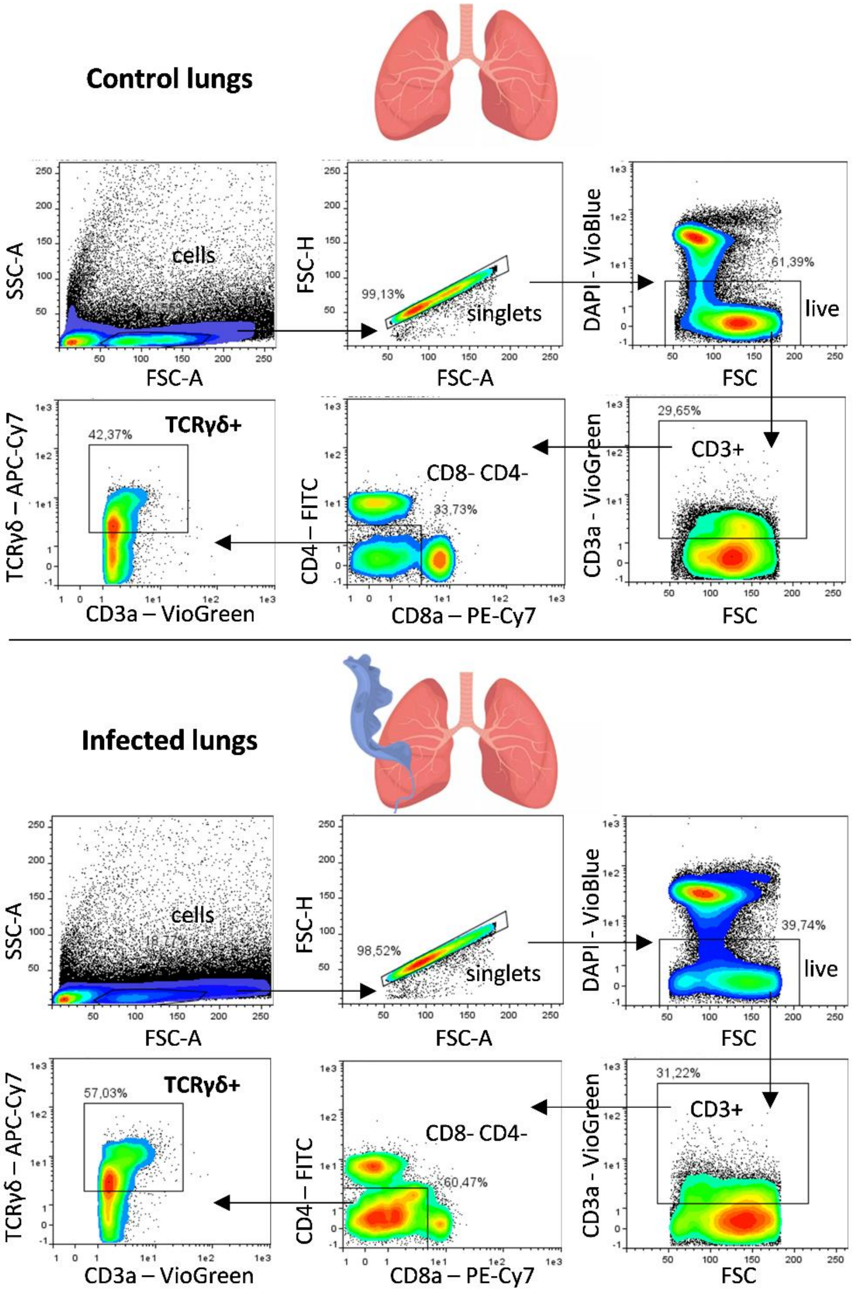

Fig. S11 Flow cytometry gating strategy for γδ T cells. A cell gate was set in an SSC-A versus FSC-A plot, followed by gating on singlets in a FSC-H versus FSC-A plot. Live ($DAPI^-$) cells were divided into two fractions: $CD3^+$ γδ T cells were further selected as being $CD8^-$ and $CD4^-$ and $TCR\gamma\delta^+$. The gating strategy is shown in control and infected lungs (21 dpi). Parts of the figure were prepared by using clipart from Servier Medical Art. Servier Medical Art by Servier is licensed under a Creative Commons Attribution 3.0 Unported License (https://creativecommons.org/licenses/by/3.0/).

Table S1. Antibody reagents used to differentiate mouse immune cells in the lungs by flow cytometry.

Cell surface marker	Fluorophore	Clone N°	Manufacturer	Dilution
CD45	VioGreen	REA737	Miltenyi Biotec	1:200
CD11b	PerCP	M1/70	BioLegend®	1:400
CD11c	APC-Cy7	N418	Miltenyi Biotec	1:100
SiglecF	PE	REA798	Miltenyi Biotec	1:50
Ly6C	APC	AL-21	BD Biosciences	1:200
Ly6G	FITC	1A8	Miltenyi Biotec	1:400
F4/80	PE-Cy7	BM8	BioLegend®	1:200
DAPI	VioBlue	/	Miltenyi Biotec	1:100
CD4	FITC	GK1.5	BioLegend®	1:250
CD8	PE-Cy7	53-6.7	Miltenyi Biotec	1:250
TCRβ	APC	H57-597	BioLegend®	1:200
NK1.1	APC-Cy7	PK136	Miltenyi Biotec	1:133
IgM	PerCP	REA979	Miltenyi Biotec	1:50
CD25	PE	REA568	Miltenyi Biotec	1:50
B220	VioGreen	RA3-6B2	Miltenyi Biotec	1:50
DAPI	VioBlue	/	Miltenyi Biotec	1:100
CD3a	Brilliant Violet 510™	145-2C11	BioLegend®	1:40
CD4	FITC	GK1.5	BioLegend®	1:250
CD8	PE-Cy7	53-6.7	Miltenyi Biotec	1:250
TCRγδ	APC-Cy7	GL3	BioLegend®	1:80
DAPI	VioBLue	/	Miltenyi Biotec	1:100

Table S2. Characteristic cell surface markers for the identification of mouse immune cells in the lungs with flow cytometry [1-4].

Cell type	Flow cytometry markers
Eosinophils	$CD45^{+}$ $CD11b^{+}$ $CD11c^{lo/-}$ $SiglecF^{+}$
Alveolar macrophages	$CD45^{+}$ $CD11b^{+}$ $CD11c^{+}$ $SiglecF^{+}$
Neutrophils	$CD45^{+}$ $CD11b^{+}$ $SiglecF^{-}$ $Ly6G^{+}$
$Ly6C^{+}$ monocytes/macrophages	$CD45^{+}$ $CD11b^{+}$ $SiglecF^{-}$ $Ly6G^{-}$ SSC^{lo} $Ly6C^{+}$
$F4/80^{+}$ macrophages	$CD45^{+}$ $CD11b^{+}$ $SiglecF^{-}$ $Ly6G^{-}$ SSC^{lo} $Ly6C^{-}$ $F4/80^{+}$
Dendritic cells	$CD45^{+}$ $CD11b^{+}$ $SiglecF^{-}$ $Ly6G^{-}$ $CD11c^{+}$
$CD4^{+}$ αβ T cells	$CD45^{+}$ $TCR\beta^{+}$ $CD8a^{-}$ $CD4^{+}$ $CD25^{-}$
$CD4^{+}/CD25^{+}$ αβ T cells	$CD45^{+}$ $TCR\beta^{+}$ $CD8a^{-}$ $CD4^{+}$ $CD25^{+}$
$CD8^{+}$ αβ T cells	$CD45^{+}$ $TCR\beta^{+}$ $CD8a^{+}$ $CD4^{-}$
NK cells	$CD45^{+}$ $TCR\beta^{-}$ $NK1.1^{+}$
IgM^{-} B cells	$CD45^{+}$ $B220^{+}$ IgM^{-}
IgM^{+} B cells	$CD45^{+}$ $B220^{+}$ IgM^{+}
$CD3^{+}$ γδ T cells	$CD3a^{+}$ $CD4^{-}$ $CD8^{-}$ $TCR\gamma\delta^{+}$

References

1 Stevens, W. W., Kim, T. S., Pujanauski, L. M., Hao, X. & Braciale, T. J. Detection and quantitation of eosinophils in the murine respiratory tract by flow cytometry. *J Immunol Methods* **327**, 63-74, doi:10.1016/j.jim.2007.07.011 (2007).

2 Misharin, A. V., Morales-Nebreda, L., Mutlu, G. M., Budinger, G. R. & Perlman, H. Flow cytometric analysis of macrophages and dendritic cell subsets in the mouse lung. *Am J Respir Cell Mol Biol* **49**, 503-510, doi:10.1165/rcmb.2013-0086MA (2013).

3 Zhao, L. *et al.* Changes of CD4+CD25+Foxp3+ regulatory T cells in aged Balb/c mice. *J Leukoc Biol* **81**, 1386-1394, doi:10.1189/jlb.0506364 (2007).

4 Bulte, D. *et al.* Miltefosine enhances infectivity of a miltefosine-resistant *Leishmania infantum* strain by attenuating its innate immune recognition. *PLoS Negl Trop Dis* **15**, e0009622, doi:10.1371/journal.pntd.0009622 (2021).
