# Supplementary material for: Impact of pulmonary African trypanosomes on the immunology and function of the lung
Source: Nat Commun. 2022 Nov 18;13:7083. doi: 10.1038/s41467-022-34757-w (PMC9674601; doi:10.1038/s41467-022-34757-w)
Supplement: Supplementary file 3 — Reporting Summary [file 41467_2022_34757_MOESM3_ESM.pdf]

nature portfolio

Corresponding author(s): Guy Caljon

Last updated by author(s): 27/10/22

Reporting Summary

Nature Portfolio wishes to improve the reproducibility of the work that we publish. This form provides structure for consistency and transparency in reporting. For further information on Nature Portfolio policies, see our Editorial Policies and the Editorial Policy Checklist.

Statistics

For all statistical analyses, confirm that the following items are present in the figure legend, table legend, main text, or Methods section.

n/a	Confirmed	
☐	☒	The exact sample size (*n*) for each experimental group/condition, given as a discrete number and unit of measurement
☐	☒	A statement on whether measurements were taken from distinct samples or whether the same sample was measured repeatedly
☐	☒	The statistical test(s) used AND whether they are one- or two-sided<br>*Only common tests should be described solely by name; describe more complex techniques in the Methods section.*
☒	☐	A description of all covariates tested
☒	☐	A description of any assumptions or corrections, such as tests of normality and adjustment for multiple comparisons
☐	☒	A full description of the statistical parameters including central tendency (e.g. means) or other basic estimates (e.g. regression coefficient) AND variation (e.g. standard deviation) or associated estimates of uncertainty (e.g. confidence intervals)
☐	☒	For null hypothesis testing, the test statistic (e.g. F, t, r) with confidence intervals, effect sizes, degrees of freedom and P value noted<br>*Give P values as exact values whenever suitable.*
☒	☐	For Bayesian analysis, information on the choice of priors and Markov chain Monte Carlo settings
☒	☐	For hierarchical and complex designs, identification of the appropriate level for tests and full reporting of outcomes
☒	☐	Estimates of effect sizes (e.g. Cohen's *d*, Pearson's *r*), indicating how they were calculated

Our web collection on statistics for biologists contains articles on many of the points above.

Software and code

Policy information about availability of computer code

Data collection	LivingImage v4.3.1, AnalySIS iTEM software (Olympus), nSolver 4.0 (NanoString)
Data analysis	Omics Playground v2.8.5, GraphPad Prism 7 and 9, FlowLogic 8.6, LivingImage v4.3.1, nSolver 4.0 (NanoString)

For manuscripts utilizing custom algorithms or software that are central to the research but not yet described in published literature, software must be made available to editors and reviewers. We strongly encourage code deposition in a community repository (e.g. GitHub). See the Nature Portfolio guidelines for submitting code & software for further information.

Data

Policy information about availability of data

All manuscripts must include a data availability statement. This statement should provide the following information, where applicable:

- Accession codes, unique identifiers, or web links for publicly available datasets
- A description of any restrictions on data availability
- For clinical datasets or third party data, please ensure that the statement adheres to our policy

The authors declare that the data underlying the findings of this study are available within the paper and its Supplementary Information files and are available upon request. The source data underlying Figs. 1, 45-10 and Supplementary Figs. 37-58 are provided as Source Data File. nCounter transcriptomics datasets have been made available at Gene Expression Omnibus (GEO accession GSE212622; https://www.ncbi.nlm.nih.gov/geo/query/acc.cgi?acc=GSE212622).

Field-specific reporting

Please select the one below that is the best fit for your research. If you are not sure, read the appropriate sections before making your selection.

☒ Life sciences ☐ Behavioural & social sciences ☐ Ecological, evolutionary & environmental sciences

For a reference copy of the document with all sections, see nature.com/documents/nr-reporting-summary-flat.pdf

Life sciences study design

All studies must disclose on these points even when the disclosure is negative.

Sample size	For all in vivo work 3-5 animals have been used in each group, time point and independent experiment. Sample size information is provided in the methods section (animals and statistics section) and in the figure legends of the corresponding experiments.<br>For the descriptive microscopy of the infected lung tissue and the nCounter transcript analysis 3 mice per group was chosen as sufficient based on our previous experience with transcriptomics and the observed variation in the infection model.<br>For the flow cytometric analysis, the required amount of mice per group was estimated using the G-power software package considering an ANOVA repeated measures, between factors analysis was performed to compare immune cells per milliliter obtained by flow cytometry of three groups (non-infected, 10 dpi and 21 dpi). The means and SD used in the effect size calculation are based on an experiment performed with 3 mice/group. The total sample size was estimated to be 9, resulting in 3 mice per group and time point.<br>For the lung function measurements, the exact amount of mice per group was calculated using the G-power software package. An ANOVA repeated measures, between factors analysis was performed to compare airway resistance obtained by flexiVent analysis of three groups (non-infected, 10 dpi and 21 dpi) with nine measurements. The means and SD used in the effect size calculation are based on an experiment performed with 3 mice/group. The total sample size was estimated to be 15 resulting in 5 mice per group and time point. This is in accordance with the sample size reported in literature of Flexivent and breath analysis measurements (Kottom et al., 2022; https://doi.org/10.1007/s40268-022-00389-0).
Data exclusions	In the flexivent experiment, 1 mouse of each group (NI, 10 dpi and 21 dpi) was excluded due to errors with the experimental setup (which led to an accumulation of fluid into the lungs) which did not allow us to acquire a complete lung function profile of these mice.
Replication	Microscopy experiments were performed in 1 independent experiment. Findings were considered relevant only if they occurred in all tested animals. nCounter analysis was performed once on 3 independent mice as well as 2 different timepoints. All flow cytometry experiments were performed in at least 2 independent repeats (except for the gamma delta T cells which was performed once as part of the rebuttal process).
Randomization	Animals were allocated in experimental groups based on simple randomization.
Blinding	Blinding was not executed for this study since it consists of a characterization of a disease phenotype and thus considered not subject to subjectivity of the researcher.

Reporting for specific materials, systems and methods

We require information from authors about some types of materials, experimental systems and methods used in many studies. Here, indicate whether each material, system or method listed is relevant to your study. If you are not sure if a list item applies to your research, read the appropriate section before selecting a response.

Materials & experimental systems

n/a	Involved in the study	
☐	☒	Antibodies
☐	☒	Eukaryotic cell lines
☒	☐	Palaeontology and archaeology
☐	☒	Animals and other organisms
☒	☐	Human research participants
☒	☐	Clinical data
☒	☐	Dual use research of concern

Methods

n/a	Involved in the study	
☒	☐	ChIP-seq
☐	☒	Flow cytometry
☒	☐	MRI-based neuroimaging

Antibodies

Antibodies used	Specific antibodies against: CD45 (VioGreen, REA737, Miltenyi Biotec, 130-110-803), CD11b (PerCP, M1/70, BioLegend®, 101229), CD11c (APC-Cy7, N418, Miltenyi Biotec, 130-107-514), SiglecF (PE, REA798, Miltenyi Biotec, 130-112-174), Ly6C (APC, AL-21, BD Biosciences, 560595), Ly6G (FITC, 1A8, Miltenyi Biotec, 130-102-934), F4/80 (PE-Cy7, BM8, BioLegend®, 123113), CD4 (FITC, GK1.5, BioLegend®, 100405), CD8 (PE-Cy7, 53-6.7, Miltenyi Biotec, 130-120-748), TCR-β (APC, H57-597, BioLegend®, 109211), B220 (VioGreen, RA3-6B2, Miltenyi Biotec, 130-118-552), NK1.1 (APC-Cy7, PK136, Miltenyi Biotec, 130-118-686), IgM (PerCP, REA979, Miltenyi Biotec, 130-116-316), CD25 (PE, REA568, Miltenyi Biotec, 130-120-766), CD3a (Brilliant Violet 510™ 145-2C11 BioLegend®, 100353), TCRγδ (APC-Cy7 GL3 BioLegend®, 118143)

	Plaque assay: palivizumab (Astra Zeneca) : leftovers provided by the department of Pediatrics, Antwerp University, goat-anti human secondary IgG conjugated with horseradish peroxidase (Invitrogen, REF 31410, Catalog # A18805)
Validation	All antibodies obtained from various companies have been validated in the past or before the start of experiments in this paper. Complete validation reports can be found on the company websites:<br>* Miltenyi biotec: https://www.miltenyibiotec.com/ for antibodies: CD45 (VioGreen, REA737, Miltenyi Biotec, 130-110-803), CD11c (APC-Cy7, N418, Miltenyi Biotec, 130-107-514), SiglecF (PE, REA798, Miltenyi Biotec, 130-112-174), Ly6G (FITC, 1A8, Miltenyi Biotec, 130-102-934), CD8 (PE-Cy7, 53-6.7, Miltenyi Biotec, 130-120-748), B220 (VioGreen, RA3-6B2, Miltenyi Biotec, 130-118-552), NK1.1 (APC-Cy7, PK136, Miltenyi Biotec, 130-118-686), IgM (PerCP, REA979, Miltenyi Biotec, 130-116-316), CD25 (PE, REA568, Miltenyi Biotec, 130-120-766)<br>* Biolegend: https://www.biolegend.com/ for antibodies: CD11b (PerCP, M1/70, BioLegend®, 101229), F4/80 (PE-Cy7, BM8, BioLegend®, 123113), CD4 (FITC, GK1.5, BioLegend®, 100405), CR-β (APC, H57-597, BioLegend®, 109211), CD3a (Brilliant Violet 510™ 145-2C11 BioLegend®, 100353), TCRγδ (APC-Cy7 GL3 BioLegend®, 118143)<br>* BD Biosciences: https://www.bdbiosciences.com/ for antibody: Ly6C (APC, AL-21, BD Biosciences, 560595)

Eukaryotic cell lines

Policy information about cell lines

Cell line source(s)	The T. b. brucei AnTAR1 and the DsRED and PpyRE9 transgenic strains were kindly provided by Nick van Reet and Philippe Buscher from the Institute of Tropical Medicine in Antwerp.<br>HEp-2 cells were obtained from ATCC.
Authentication	All T. b. brucei strains were authenticated by SL-RNA qPCR (doi: 10.1016/j.jmoldx.2014.02.006). Bioluminescence was validated using bioluminescent imaging and the DsRED phenotype was validated using flow cytometry, always in comparison with the non-transfected parent strain.
Mycoplasma contamination	Parasite and cell cultures were free of Mycoplasma.
Commonly misidentified lines (See ICLAC register)	No commonly misidentified lines were used in this study.

Animals and other organisms

Policy information about studies involving animals; ARRIVE guidelines recommended for reporting animal research

Laboratory animals	C57BL/6JRj mice were used, all female between 6-8 weeks at the start of infection.<br>Male and female tsetse flies (Glossina morsitans morsitans) were used in this study. Newly emerged flies were infected and used after 3-4 weeks post infection for natural transmission to the mouse host. Infected flies were used throughout their entire lifespan.
Wild animals	No wild animals were used in this study.
Field-collected samples	No field-collected samples were used in this study.
Ethics oversight	Ethical Committee (ECD) of the University of Antwerp. Ethical approval: UA-ECD 2017–04 and 2022–55

Note that full information on the approval of the study protocol must also be provided in the manuscript.

Flow Cytometry

Plots

Confirm that:

☒ The axis labels state the marker and fluorochrome used (e.g. CD4-FITC).

☒ The axis scales are clearly visible. Include numbers along axes only for bottom left plot of group (a 'group' is an analysis of identical markers).

☒ All plots are contour plots with outliers or pseudocolor plots.

☒ A numerical value for number of cells or percentage (with statistics) is provided.

Methodology

Sample preparation	To generate single-cell suspensions from perfused lungs, the Mouse Lung Dissociation Kit (Miltenyi Biotec) was used. Briefly, resected lung lobes were transferred into gentleMACSTM C tubes (Miltenyi Biotec) containing 2.4 mL of the enzyme mix (Miltenyi Biotec). After running a first lung-specific gentleMACSTM program, samples were incubated for 30 min in a 37°C water bath, whilst shaken every 5 min, followed by a second lung-specific gentleMACSTM program. Next, cell suspensions were transferred into Falcon tubes and centrifuged for 10 min at 300×g (4°C), and pellets were resuspended in 3 mL ammonium-chloride-potassium (ACK) buffer (0.15 M NH4Cl, 1.0 mM KHCO3, 0.1 mM Na2EDTA) for a 7 min erythrocyte lysis at RT. Pellets were recovered after a centrifugation step at 300×g (4°C), resuspended in PBS + 0.2% bovine serum albumin (BSA) buffer and filtered through a 100 µm filter (Miltenyi Biotec). Cell suspensions were counted using a KOVA® counting chamber. Cell suspensions (2 × 107/mL) were treated with FcɣR-blocking agent (anti-CD16/32, clone 2.4G2, BD Biosciences)

	for 15 min in PBS + 0.2% BSA buffer. Next, cells were incubated for 20 min at 4°C with a mix of fluorescent conjugated anti-mouse antibodies at optimized concentrations (Table S1). DAPI Staining Solution (Miltenyi Biotec) was used to assess viability.
Instrument	MACSQuant Analyzer 10 (Miltenyi Biotec) serienummer: 21316
Software	FlowLogic Software (Miltenyi Biotec)
Cell population abundance	No cell sorting was performed during this study.
Gating strategy	Gating strategy is provided in the supplementary information, briefly for myeloid cells: a cell gate was set in an SSC-A versus FSC-A plot, followed by gating on singlets in an FSC-H versus FSC-A plot. Live (DAPI-) CD45+ cells were divided into two fractions: SiglecF+ cells were further characterized as alveolar macrophages (CD11c+) and eosinophils (CD11clo/-) and SiglecF- cells were further identified as neutrophils (CD11b+, Ly-6G+), monocytes (CD11b+, Ly6G-, SSClo, Ly6Chi), macrophages (CD11b+, Ly6G-, SSClo, Ly6C-, F4/80+) and dendritic cells (CD11b+, Ly6G-, CD11c+).<br>For lymphoid cells: a cell gate was set in an SSC-A versus FSC-A plot, followed by gating on singlets in a FSC-H versus FSC-A plot. Live (DAPI-) cells were divided into two fractions: B220+ cells were further characterized as IgM+ B cells (IgM+) and IgM- B cells (IgM-) and B220- cells were further identified as natural killer cells (TcR-β-, NK1.1+), CD8+ T cells (TcR-β+, CD8+), CD4+ T cells (TcR-β+, CD8- CD4+) and CD4+/CD25+ T cells (TcR-β+, CD8-, CD4+, CD25+).

☒ Tick this box to confirm that a figure exemplifying the gating strategy is provided in the Supplementary Information.
